# The Educational Inclusion of Students with Autism Spectrum Disorder: Teachers’ Feelings, Attitudes, and Concerns About Inclusion in Spain

**DOI:** 10.3390/ejihpe15100200

**Published:** 2025-09-29

**Authors:** Alejandra Bolado Peña, Félix Menéndez-Vega, Steven Van Vaerenbergh, Mercedes Arias-Pastor, Jerónimo J. González-Bernal

**Affiliations:** 1 Ministry of Education, Vocational Training and Universities of the Government of Cantabria, 39005 Santander, Spain; alejandra.bolado@educantabria.es; 2 Department of Health Sciences, University of Burgos, 09001 Burgos, Spain; jejavier@ubu.es; 3 Department of Mathematics, Statistics and Computation, University of Cantabria, 39005 Santander, Spain; steven.vanvaerenbergh@unican.es; 4 Department of Education, University of Cantabria, 39005 Santander, Spain; mercedes.ariaspastor@unican.es

**Keywords:** educational inclusion, Autism Spectrum Disorder, secondary school teachers, attitudes, concerns, feelings

## Abstract

**Introduction:** The educational inclusion of students with Autism Spectrum Disorder (ASD) in Spain has been promoted through regulations such as LOMCE and LOMLOE. However, its effective implementation depends on teachers’ attitudes and perceptions. This study analyzes teachers’ feelings, attitudes, and concerns regarding the inclusion of students with ASD. **Methods:** A quantitative, descriptive, and cross-sectional study was conducted with a sample of 2310 teachers from different educational stages in Spain. The SACIE-R and INTEA questionnaires were used to assess teachers’ perceptions of inclusion. ANOVA tests and Spearman correlations were applied for statistical analysis. **Results:** The results show that the variable “Attitudes” follows a normal distribution, indicating a stable perception of inclusion. In contrast, the variables “Feelings” and “Concerns” present an inverse relationship: the greater the concern, the fewer positive feelings toward inclusion. Significant differences were found based on gender, type of school, educational stage, and teaching specialty. **Discussion:** Positive attitudes toward inclusion are associated with greater training and specialization in diversity. Special Education teachers show better perceptions, while in Secondary Education, concerns and negative feelings prevail. Continuous training emerges as a key factor in improving teachers’ perceptions. **Conclusions:** The study highlights the importance of training programs and support strategies to promote effective inclusion. Strengthening support networks and teacher training is recommended to improve attitudes toward the inclusion of students with ASD.

## 1. Introduction

Autism Spectrum Disorder (ASD) is a neurodevelopmental condition characterized by persistent difficulties in social communication and interaction, along with restricted and repetitive behaviors and interests (United Nations, 2006). These features manifest in diverse ways and intensities, which makes ASD a highly heterogeneous condition. In the school context, students with ASD may face challenges in participating in group dynamics, understanding implicit social rules, or adapting to sudden changes in routines. Such difficulties are often exacerbated when classrooms are structured around uniform teaching strategies that do not consider individual learning profiles. Research has shown that, in addition to these individual challenges, structural and attitudinal barriers within schools—such as stigma, lack of awareness, or insufficient training among teachers—can hinder the full educational participation of students with ASD (Lindsay et al., 2014; Morris et al., 2023). For this reason, the concept of inclusive education emphasizes not only the physical presence of students in regular classrooms but also their active engagement, participation, and development in equitable learning environments (Ainscow, 2020).

Spain has progressively aligned its educational policies with international standards for inclusive education, such as those established in the Salamanca Statement minister (Ministry of Education and Science Spain, 1994) and the Convention on the Rights of Persons with Disabilities (United Nations, 2006). The Organic Law of Education (LOE) was a landmark in recognizing the right of students with special educational needs to receive appropriate support within the mainstream school system (Gobierno de España, 2006). Subsequently, the Organic Law for the Improvement of Educational Quality (LOMCE) introduced reforms intended to promote equity and adapt educational provision to the diversity of students, while the Organic Law on Education (LOMLOE) reinforced inclusive principles, placing emphasis on individualized responses and reasonable accommodations to ensure participation and learning (Gobierno de España, 2006, 2013). These successive laws reflect a gradual but steady commitment to fostering inclusion, although their implementation continues to be uneven across different regions and educational stages.

Beyond legislation, the Spanish education system has introduced various provisions to support students with ASD. These include curricular adaptations, flexible grouping, and individualized support plans that adjust the curriculum to specific learning needs (Vidal-Esteve & Kossyvaki, 2024). Many schools have developed support classrooms for students with ASD (Aulas TEA), where students receive specialized assistance while remaining integrated in mainstream settings for much of their daily school life (Vela Llauradó et al., 2020). Moreover, specialized interdisciplinary teams comprising psychologists, guidance counselors, speech therapists, and special education teachers work in collaboration with general education staff to design and implement inclusive strategies (Muñoz-Martínez et al., 2023). In some cases, co-teaching models allow general and special education teachers to jointly plan and deliver instruction, thereby enhancing differentiation and reducing barriers to participation (King-Sears et al., 2021). The increasing use of assistive technologies such as communication devices, visual schedules, or digital platforms also plays a significant role in supporting autonomy and learning for students with ASD (Khowaja et al., 2020). Despite these efforts, however, resource limitations, uneven teacher training, and variability in professional collaboration remain critical challenges (Romero-Contreras et al., 2021).

Teachers’ attitudes are a decisive factor in determining whether inclusive provisions translate into effective practices. Positive attitudes have been consistently linked to a greater willingness to adapt instruction and engage in collaborative practices, while negative or ambivalent perceptions often act as significant barriers to inclusion (Forlin et al., 2011). In Spain, research indicates variability depending on school stage and professional background. Primary education teachers often report more favorable attitudes toward inclusion, whereas secondary school teachers are more likely to express concerns about classroom management, curricular demands, or a lack of training (Gómez-Marí et al., 2022; Triviño-Amigo et al., 2023). Internationally, similar patterns have been observed: in the UK found that secondary teachers expressed greater attitudinal barriers (Humphrey & Symes, 2013), while Israel and the USA demonstrated that targeted training programs can significantly improve teachers’ perceptions and self-efficacy in working with students with ASD (Crispel & Kasperski, 2021; Morrier et al., 2011). These findings suggest that training, school culture, and institutional support are essential factors shaping teachers’ readiness for inclusive practice.

Despite the importance of this issue, there is still limited large-scale quantitative research in Spain that systematically analyzes how teachers’ attitudes, perceptions, and training affect the inclusion of students with ASD across different educational stages and institutional contexts. Most existing studies focus on small samples or single school levels, which limits the generalizability of findings. Furthermore, comparisons between mainstream and special education settings are scarce, even though such comparisons can provide valuable insights into the profiles of teachers who encounter greater attitudinal barriers and those who develop more inclusive practices.

The present study aims to address this gap by analyzing the feelings, attitudes, and concerns of a large sample of 2310 teachers from different educational stages in Spain regarding the inclusion of students with ASD. This research contributes to the existing literature by identifying teacher profiles associated with attitudinal barriers and highlighting factors that may guide the design of professional training and intervention programs. The central research question is: *How do teachers’ attitudes, perceptions, and training influence the inclusion of students with ASD in Spanish schools across different educational stages and institutional contexts?* Based on previous evidence, we hypothesize that (1) teachers’ attitudes toward inclusion will differ significantly by educational stage, with secondary teachers reporting greater barriers; (2) teachers in special education centers will display more consolidated inclusive practices compared to mainstream teachers; and (3) higher levels of specific training will be associated with more positive attitudes toward inclusion.

## 2. Materials and Methods

### 2.1. Study Design

This study is framed as a quantitative, descriptive, cross-sectional, and multicentric study, conducted in various educational institutions across all autonomous communities of Spain. Its objective is to analyze the feelings, attitudes, and concerns regarding the inclusion of students with Autism Spectrum Disorder (ASD).

Sampling was non-probabilistic, convenience-based, and voluntary, since it was not feasible to access a complete census of teachers in the Spanish educational system. Although random sampling is methodologically more robust, Gall (2006) argued that ‘it is better to conduct a study with a convenience sample than not to conduct any study at all, provided that the sample fits the purpose of the research’ (Gall, 2006). In this regard, developmental science research has confirmed that this sampling strategy is the norm, being present in 92.5% of the studies reviewed, given its practicality and efficiency (Bennett et al., 1984).

### 2.2. Population

The sample consisted of 2310 teachers from different educational stages: early childhood education, primary education, compulsory secondary education, high school, vocational training, basic compulsory education (EBO), and transition to adult life programs (TVA).

Three types of institutions were included: mainstream schools, special education schools and mainstream schools with special education classrooms and other educational programs (such as adapted vocational training or transition programs). The inclusion of these different types of institutions was intended to compare teachers’ perceptions across diverse educational settings. While some approaches consider special education schools to be segregating models, their inclusion in this study was justified by the aim of contrasting their teachers’ perspectives with those from mainstream schools and examining how institutional context influences attitudes toward inclusion. This design provides a more nuanced picture of the Spanish educational reality.

The sample also included teachers from public (72%), semi-private (22%), and private (6%) schools, thereby representing different types of school ownership. Data were collected from all autonomous communities, although a larger proportion of responses was concentrated in regions such as the Valencian Community and Andalusia, which limits the representativeness of the findings.

All teachers participated voluntarily, providing the necessary information for the collection of demographic data. Ethical and privacy principles were respected, and all participants gave their informed consent to be included in this study. The study adhered to the ethical principles outlined in the Declaration of Helsinki and was approved by the Clinical Research Ethics Committee of the University of Burgos, reference IO-10/2024, on 21 February 2024.

### 2.3. Procedure

Data collection was carried out through an indirect distribution method: the online survey link, accompanied by an invitation letter, was sent to school management teams, who then disseminated it among their teaching staff through internal networks and institutional emails.

Although this strategy facilitated wide geographic coverage and the participation of a large number of teachers, it prevented the calculation of the exact response rate. Moreover, it likely introduced a self-selection bias, since teachers with greater interest in inclusive education or with prior experience in teaching students with ASD may have been more inclined to participate.

### 2.4. Instruments

Three questionnaires were used, all adapted to the Spanish context:

1. SACIE-R (Sentiments, Attitudes, and Concerns about Inclusive Education–Revised) (Forlin et al., 2011). This instrument consists of 15 items on a 4-point Likert scale (1 = strongly disagree; 4 = strongly agree). It evaluates teachers’ sentiments, attitudes, and concerns regarding inclusion. Factor analysis confirmed the presence of three distinct dimensions: Attitudes (5 items, α = 0.86) indicating strong internal consistency, Feelings (5 items, α = 0.63) suggesting moderate reliability but adequate for exploratory studies, and Concerns (5 items, α = 0.71) reflecting acceptable consistency for research use. This factorial structure has been reported in previous validation studies (Gómez-Marí et al., 2022; Triviño-Amigo et al., 2023), and it was deemed more appropriate to analyze these subscales independently rather than as a single total score.
2. A modified version of AREISA (Attitudes and Readiness towards Educational Inclusion of Students with Autism). The original questionnaire comprises 22 items on a 4-point Likert scale. It measures teachers’ general attitudes toward the inclusion of students with specific educational needs.
3. INTEA (Inclusive Education for Students with Autism). Developed from the AREISA, the INTEA specifically measures teachers’ attitudes toward the inclusion of students with ASD in mainstream classrooms. It consists of 10 items; however, in this study only 3 showed high internal consistency (α = 0.95), and therefore these items were used in the main analyses.

The three instruments were applied in a complementary manner: SACIE-R provided a broad evaluation of inclusive education, while INTEA focused specifically on ASD-related perceptions. AREISA served as the conceptual basis for the adaptation, supporting triangulation.

The full set of items from each questionnaire is presented in Appendix A and Supplementary S1, in order to enhance methodological transparency.

### 2.5. Statistical Analysis

Statistical analysis was conducted using SPSS software version 28 (IBM-Inc., Chicago, IL, USA).

Although the data from the studied variables did not follow a normal distribution, parametric ANOVA tests were performed, following the study by Blanca M. et al., which provides empirical evidence supporting the robustness of ANOVA tests even when dealing with non-normally distributed variables (Blanca et al., 2017).

Therefore, parametric ANOVA tests were applied in the study under to compare the means of feelings, attitudes, and concerns across different teaching specializations regarding the inclusion of students with ASD.

In the statistical analysis of the SACIE-R questionnaire dimensions, it was observed that the “Attitudes” factor follows a normal distribution, indicating that its values are symmetrically distributed around the mean. Higher scores in this dimension reflect more positive attitudes toward inclusive education.

Conversely, the variables “Feelings” and “Concerns” show an inverse relationship, meaning that as one increases, the other decreases, and vice versa. Consequently, higher scores in these dimensions indicate less favorable perceptions of inclusive education.

## 3. Results

Three multiple linear regression analyses were conducted to explore the extent to which teachers’ sociodemographic and professional variables—such as gender, type of school, educational stage, teaching specialty, and approximate hours of training—explain attitudes toward the inclusion of students with ASD, as assessed through the dimensions of the INTEA and SACIE-R questionnaires.

Regarding the INTEA instrument, all three models showed low adjusted R^2^ values. Low adjusted R^2^ values with significant predictors suggest authentic but modest relationships, consistent with the complexity of human attitudes and the large variability in the data.

For Dimension F1 (Philosophical Issues), the model explained 1% of the variance (R^2^ = 0.010). Only the type of school showed a significant relationship (B = −0.066, *p* < 0.001), indicating that teachers in mainstream schools tend to have more favorable perceptions about the principles of inclusion compared to those working in special education centers. Teaching specialty was also significant (B = 0.018, *p* = 0.018), suggesting that professional background influences these attitudes.

For Dimension F2 (Benefits of Inclusion), the model explained 0.8% of the variance (R^2^ = 0.008). Again, type of school was the only significant predictor (B = −0.047, *p* = 0.004), confirming the same trend.

In the case of the total INTEA score, the model explained 0.9% of the variance (R^2^ = 0.009). Type of school was once again significant (B = −0.056, *p* < 0.001), and teaching specialty showed a marginal trend (B = 0.013, *p* = 0.051). Specialties included Early Childhood and Primary Tutors, Secondary Education Teachers, Specialists in Attention to Diversity, and Specialists in Educational Guidance.

The remaining variables—gender, educational stage, and hours of training—were not significant in any of the models.

The multiple linear regression analysis applied to the three dimensions of the SACIE-R questionnaire aimed to identify the extent to which sociodemographic variables predict teachers’ attitudes, feelings, and concerns regarding the inclusion of students with ASD.

For Factor 1: Attitudes, the model showed an adjusted R^2^ of 0.007, indicating that the included variables explained only 0.7% of the variance. Despite the low explanatory power, some significant predictors were identified. Educational stage was negatively related to inclusive attitudes (B = −0.044, *p* = 0.004), with more favorable attitudes in lower stages. Teaching specialty was also significant (B = 0.021, *p* = 0.006), reflecting better attitudes among teachers specialized in attention to diversity. Additionally, the number of training hours showed a positive association with attitudes (B = 0.00003, *p* = 0.036), reinforcing the role of training as a facilitating factor.

For Factor 2: Feelings, the model showed an adjusted R^2^ of 0.026, making it the dimension with the highest explained variance. The results indicated that gender was a significant and negative predictor (B = −0.154, *p* < 0.001), reflecting that women express more positive feelings toward inclusion. Type of school also showed a significant negative relationship (B = −0.050, *p* < 0.001), suggesting greater emotional predisposition in special education settings. Finally, training hours were negatively associated with feelings (B = −0.00003, *p* = 0.019), indicating that more training may help reduce emotional barriers or insecurities.

For Factor 3: Concerns, the model explained 2.1% of the variance (adjusted R^2^ = 0.021). Again, type of school was a significant negative predictor (B = −0.090, *p* < 0.001), indicating that concerns are lower in specialized centers. Educational stage had a significant positive effect (B = 0.044, *p* = 0.010), with higher concerns at upper educational levels. Gender also had a significant influence (B = −0.085, *p* = 0.012), with women reporting fewer concerns. Additionally, training hours were negatively associated with concerns (B = −0.00006, *p* < 0.001), confirming that ongoing training is key to mitigating teachers’ insecurities regarding inclusion.

In summary, although the explanatory power of the models is limited, consistent patterns emerge in which school type, gender, educational stage, and teacher training stand out as key factors influencing attitudes and perceptions toward the inclusion of students with ASD.

### 3.1. Mann–Whitney U Test for Comparing Gender in the SACIE-R and INTEA Questionnaires

In Table 1, the Mann–Whitney U test shows significant differences between genders in the “Feelings” and “Concerns” dimensions of the SACIE-R instrument, (U = −6.838, *p* < 0.001) and (U = −3.652, *p* < 0.001), respectively. It can be observed that the mean rank for women is lower than for men, indicating that women exhibit more positive feelings and fewer concerns regarding inclusion.

However, no significant differences between genders are found in the “Philosophical Issues,” “Benefits of Inclusion,” and “Total INTEA Score” dimensions of the INTEA instrument.

### 3.2. ANOVA Test for Comparing School Type and the SACIE-R and INTEA Questionnaires

Table 2 presents the ANOVA analysis for different types of schools and the SACIE-R instrument scores, revealing significant differences between groups in the “Feelings” (*p* < 0.001) and “Concerns” (*p* < 0.001) dimensions. Post hoc analyses show that Special Education Centers present significant differences compared to the other groups, with lower mean scores. This indicates that these centers express more positive feelings and lower levels of concern regarding inclusion.

Similarly, the ANOVA analysis for different types of schools and the INTEA questionnaire scores reveals significant differences between groups in the “Benefits of Inclusion” dimension (*p* < 0.001), as well as in “Philosophical Issues” and the “Total INTEA Score” (*p* < 0.001). Post hoc analyses indicate that Mainstream Schools show significant differences compared to Schools with Special Education Classrooms (*p* < 0.001) and Special Education Centers (*p* = 0.010) in Factor 1 of the INTEA. With a higher mean, these schools reflect a more favorable perspective on philosophical issues related to inclusion.

Regarding the “Benefits of Inclusion” dimension, Mainstream Schools also show significant differences compared to Schools with Special Education Classrooms (*p* = 0.001) and Special Education Centers (*p* = 0.006), indicating a higher perception of the benefits of inclusion.

Finally, Mainstream Schools obtain a higher total score on the INTEA compared to Schools with Special Education Classrooms (*p* < 0.001) and Special Education Centers (*p* = 0.004), reflecting a more positive overall perception of inclusion.

### 3.3. ANOVA Test for Comparing Educational Stage and the SACIE-R and INTEA Questionnaires

In Table 3, the ANOVA analysis for different educational stages using the SACIE-R instrument reveals significant differences in all three factors (*p* < 0.001). In the post hoc analyses for the “Attitudes” dimension, Primary Education shows significant differences compared to Early Childhood Education and Basic Obligatory Education (E.B.O) (*p* = 0.005), Secondary Education (*p* < 0.001), and Transition to Adulthood (T.V.A) (*p* = 0.031). With a higher mean score in this dimension, it indicates more positive attitudes toward inclusion. In the post hoc analyses for “Feelings,” Secondary Education shows significant differences compared to Early Childhood Education (*p* = 0.021), Primary Education (*p* = 0.014), and T.V.A (*p* < 0.001), as well as a trend towards significance compared to E.B.O (*p* = 0.055). With a higher mean score than the other groups, Secondary Education presents the worst feelings towards inclusion. Additionally, T.V.A shows significant differences compared to Early Childhood Education and Primary Education (*p* = 0.005), and Secondary Education (*p* < 0.001). T.V.A has better feelings toward inclusion as it has a lower mean score. Regarding the post hoc analyses for “Concerns,” Secondary Education shows significant differences compared to Early Childhood Education, Primary Education, and T.V.A (*p* < 0.001), and E.B.O (*p* = 0.009). Secondary Education has a higher mean score, indicating more concerns regarding inclusion. Furthermore, T.V.A shows significant differences compared to Early Childhood Education (*p* = 0.027), Primary Education (*p* = 0.012), and Secondary Education (*p* < 0.001), having better concerns towards inclusion as it has a lower mean score.

The table also shows the ANOVA analysis for different educational stages using the INTEA instrument, finding no significant differences in the “Philosophical Issues” dimension (*p* = 0.193), “Benefits of Inclusion” (*p* = 0.065), and the total INTEA score (*p* = 0.193).

**Figure 1:**
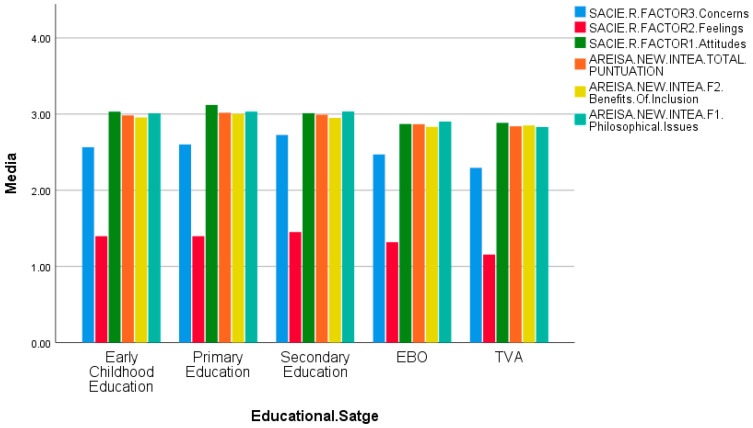
Mean inclusive attitudes of teachers by educational stage (ANOVA).

Figure 1 shows the explanatory bar chart corresponding to Table 3.

### 3.4. ANOVA Test for Comparing Teaching Specialty and the SACIE-R and INTEA Questionnaires

In Table 4, the ANOVA analysis for different teaching specialties using the SACIE-R instrument reveals significant differences in all three factors (*p* < 0.001). In the post hoc analyses of Factor 1 “Attitudes,” the Educational Guidance Specialist shows significant differences compared to the rest of the groups (*p* < 0.001), having better attitudes toward inclusion as it shows a higher mean. The Diversity Attention Specialist shows significant differences compared to the Early Childhood Education Tutor and Secondary Education Teacher (*p* < 0.001), and the Early Childhood and Primary Education Specialist Tutor (*p* = 0.003). Having a higher mean than these groups, it shows better attitudes toward inclusion. The Management Team shows significant differences compared to the Early Childhood Education Tutor (*p* < 0.001), the Early Childhood and Primary Education Specialist Tutor (*p* = 0.048), and the Secondary Education Teacher (*p* = 0.001). As the group with the highest mean, the Management Team has better attitudes toward educational inclusion. Finally, the Primary Education Tutor shows significant differences compared to the Early Childhood Education Tutor (*p* < 0.001) and the Secondary Education Teacher (*p* = 0.005), having a higher mean and thus better attitudes toward inclusion. In the post hoc analyses of Factor 2 “Feelings,” the Diversity Attention Specialist shows significant differences compared to the rest of the groups (*p* < 0.001), except for the Educational Guidance Specialist. Being the group with the lowest mean, the Diversity Attention Specialist has the best feelings toward inclusion. The Educational Guidance Specialist follows closely, showing significant differences compared to the rest of the groups (*p* < 0.001), and presents a significant difference with the Management Team (*p* = 0.019). In the post hoc analyses of Factor 3 “Concerns,” the Diversity Attention Specialist shows significant differences compared to the Educational Guidance Specialist (*p* = 0.008) and the rest of the groups (*p* < 0.001). As the group with the lowest mean, it shows better concerns toward inclusion. The Educational Guidance Specialist also shows significant differences compared to the rest of the groups, having the lowest mean and therefore better concerns toward inclusion. The Secondary Education Teacher shows significant differences compared to the Early Childhood Education Tutor (*p* = 0.002), the Primary Education Tutor (*p* = 0.022), and the Management Team (*p* < 0.001), having the worst concerns toward inclusion due to the highest mean. Moreover, the Early Childhood and Primary Education Specialist shows worse concerns toward inclusion compared to the Early Childhood Education Tutor (*p* = 0.047) and the Management Team (*p* = 0.002). Finally, the Management Team shows a trend towards significance compared to the Primary Education Tutor (*p* = 0.054), with a lower mean, indicating better concerns toward inclusion.

The table also shows the ANOVA analysis for different teaching specialties using the INTEA instrument, revealing significant differences in both factors and the total score of the questionnaire (*p* < 0.001). In the post hoc analyses of Factor 1 “Philosophical Issues,” the Educational Guidance Specialist shows significant differences compared to the rest of the groups (*p* < 0.001), having better philosophical views toward inclusion due to a higher mean. The Diversity Attention Specialist also shows significant differences compared to the Early Childhood Education Tutor (*p* < 0.001), the Primary Education Tutor (*p* = 0.008), the Early Childhood and Primary Education Specialist (*p* = 0.003), the Secondary Education Teacher (*p* = 0.006), and the Management Team (*p* = 0.036). With a higher mean, it shows better philosophical views toward inclusion. In the post hoc analyses of Factor 2 “Benefits of Inclusion,” the Educational Guidance Specialist shows significant differences compared to the Diversity Attention Specialist (*p* = 0.003) and the rest of the groups (*p* < 0.001). The Diversity Attention Specialist also shows significant differences compared to the Early Childhood Education Tutor, the Early Childhood and Primary Education Specialist, and the Secondary Education Teacher (*p* < 0.001), the Primary Education Tutor (*p* = 0.025), and the Management Team (*p* = 0.036). With a higher mean, it shows better philosophical views toward inclusion. On the other hand, the Secondary Education Teacher shows significant differences compared to the Primary Education Tutor (*p* = 0.040) and the Management Team (*p* = 0.014), having a lower mean, indicating worse benefits toward inclusion. Finally, in the post hoc analyses of the total INTEA score, the Educational Guidance Specialist shows significant differences compared to the rest of the groups (*p* < 0.001), having better attitudes toward inclusion due to a higher mean. The Diversity Attention Specialist also shows significant differences compared to the Early Childhood Education Tutor, the Early Childhood and Primary Education Specialist, and the Secondary Education Teacher (*p* < 0.001), the Primary Education Tutor (*p* = 0.009), and the Management Team (*p* = 0.025). The Management Team shows a trend toward significance compared to the Early Childhood Education Tutor and the Early Childhood and Primary Education Specialist, having a higher mean and better total scores on the questionnaire.

**Figure 2:**
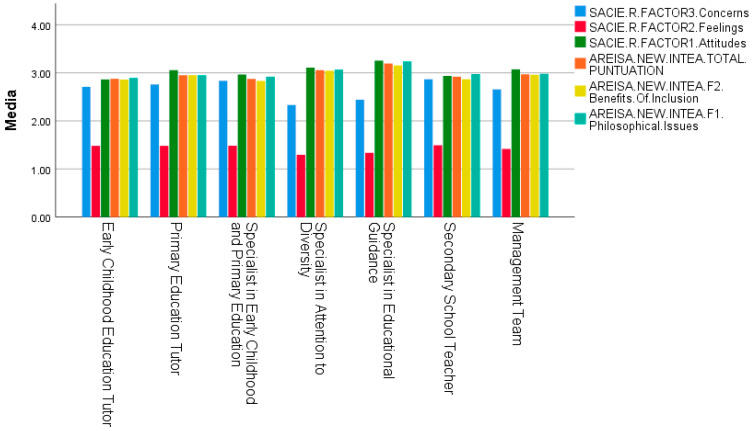
Mean inclusive attitudes of teachers by teaching specialty (ANOVA).

Figure 2 shows the explanatory bar chart corresponding to Table 4.

### 3.5. Correlation Between Training Tours and the SACIE-R and INTEA Questionnaires

Table 5 presents Spearman correlations between teachers’ training hours and SACIE-R dimensions, all significant at *p* < 0.001. Although weak in magnitude, the results indicate that more training is associated with more positive attitudes and reduced concerns. Similarly, correlations with the INTEA scale suggest that greater training is linked to more favorable philosophical perspectives and perceived benefits of inclusion.

The table also shows the significant Spearman correlation between the scores of the INTEA instrument and the training hours of the teachers (*p* < 0.001). The analysis reveals a weak correlation, suggesting that as the number of training hours increases, the teachers’ philosophical issues and benefits toward inclusion also improve.

### 3.6. Correlation Between SACIE-R and INTEA Questionnaires

Table 6 shows the significant Spearman correlation between the dimensions of the SACIE-R and INTEA instruments, with significant differences found between them (*p* < 0.001). The “Attitudes” dimension of the SACIE-R questionnaire shows a positive correlation with the dimensions and total score of the INTEA, meaning that better attitudes correspond to better philosophical issues and benefits for inclusion. On the other hand, the “Feelings” and “Concerns” dimensions show a negative correlation with the dimensions and total score of the INTEA, indicating that worse scores in feelings and concerns correspond to better results in philosophical issues and benefits for inclusion.

## 4. Discussion

The purpose of this study was to explore teachers’ attitudes, feelings, and concerns regarding the inclusion of students with ASD in Spain, analyzing the influence of gender, school type, educational stage, specialty, and training. The discussion below interprets these results in light of previous research and the practical implications for inclusive education. This study highlights the importance of teachers’ predispositions and tendencies toward inclusion, showing the attitudes, feelings, concerns, ideas, and perceived benefits that educators believe inclusion brings to students with ASD.

Firstly, women exhibit more positive feelings and fewer concerns about inclusion than men, a result that is consistent with (Jury et al., 2021), who reported that female teachers tend to show more favorable attitudes than their male counterparts. This aligns with other studies indicating similar trends (Gómez-Marí et al., 2022; Kisbu-Sakarya & Doenyas, 2021). However, regarding philosophical perceptions or perceived benefits of inclusion, no significant gender differences were found. This finding indicates that variables such as training content, the quality of professional experience, and the institutional climate may play a more decisive role than gender itself in shaping these dimensions. For example, Lindsay et al. (2014) emphasize that structured training and exposure to inclusive practices are stronger predictors of teachers’ perceptions than demographic variables (Lindsay et al., 2014).

The type of educational center influences attitudes toward inclusion. Although Special Education Centers appeared to show more positive feelings and fewer concerns, this finding must be interpreted with caution. Teachers in these schools usually work with more homogeneous groups of students and therefore may feel more confident in their teaching context. However, inclusion is only fully achieved in mainstream schools, where diversity is present and where attitudes toward difference are truly tested and developed (Watkins et al., 2019). In contrast, Mainstream Schools score higher in dimensions related to philosophical issues and benefits of inclusion, possibly due to their greater exposure to heterogeneous environments and inclusive policies that enrich their perspective (Rosen et al., 2021; Watkins et al., 2019).

Within schools, attitudes toward inclusion vary by educational stage. Secondary Education shows less positive feelings and greater concerns, possibly influenced by the typical challenges of this stage, such as varying skill levels, lack of support networks, and limited resources (Richter et al., 2020; Triviño-Amigo et al., 2022). In contrast, the Transition to Adult Life (TAL) stage reflects more favorable attitudes, likely due to its focus on addressing the individual needs of students (Crispel & Kasperski, 2021). The results also revealed important insights from teachers in early childhood and primary education. In these stages, attitudes and feelings toward inclusion were more positive, and concerns were lower compared to secondary education. This trend may be related to smaller class sizes, closer family–school collaboration, and a stronger tradition of individualized attention at these levels. Addressing these early years is essential, as they lay the foundation for inclusive practices that continue throughout schooling.

Furthermore, teaching specialties also play a key role in perceptions of inclusion. Specialists in Educational Guidance and Attention to Diversity stand out for their more positive attitudes, feelings, and concerns, a direct result of their frequent contact with students with special educational needs and the training they have received (Josilowski & Morris, 2019). It should also be recognized that general education teachers in mainstream classrooms are expected to have frequent contact with students with ASD, particularly when co-teaching models and differentiated instruction are implemented. This perspective reinforces the importance of preparing all teachers, not only specialists, to address diversity in their daily practice. In contrast, Secondary Education teachers tend to show less favorable attitudes. According to the literature, this may be due to a more rigid curriculum structure as students advance and the ongoing stigma in higher education settings for neurodivergent individuals (Aguirre et al., 2021; Azevedo et al., 2024).

In light of the results, ongoing professional development is shown to be a key factor in shaping teachers’ attitudes, feelings, and concerns about inclusion. The more time dedicated to training, the more inclusive the teachers’ attitudes become, with training acting as a facilitator for improving inclusive education (Triviño-Amigo et al., 2022). This finding is consistent with international evidence demonstrating that professional development enhances not only pedagogical knowledge but also teachers’ beliefs and attitudes toward inclusion. Studies have shown that sustained training opportunities significantly improve classroom practices and students’ academic and social outcomes (Kisbu-Sakarya & Doenyas, 2021; Pattiasina et al., 2024). However, teacher training is not the only explanation. Other factors identified in the literature include the degree of institutional support, peer collaboration, access to resources, and the existence of inclusive leadership in (Avramidis & Norwich, 2002; Triviño-Amigo et al., 2023). Additionally, cultural expectations and parental involvement have been shown to moderate teachers’ concerns and attitudes (Muñoz-Martínez et al., 2023). A comprehensive analysis should therefore consider these contextual influences together with individual training variables.

The multiple linear regression analyses confirm these relationships, showing that variables such as school type, educational stage, teaching specialty, gender, and training hours have modest but significant effects on the different dimensions studied (attitudes, feelings, concerns, philosophical issues, and perceived benefits). For example, continuous training is positively associated with better attitudes and philosophical perspectives on inclusion, while school type and teaching specialty influence outcomes differently depending on the dimension. These results reinforce the idea that multiple contextual and personal variables interact to shape teachers’ predisposition toward the educational inclusion of students with ASD.

The relationship between the SACIE-R and INTEA questionnaires supports the convergent validity of the findings: more positive attitudes are associated with greater recognition of the benefits of inclusion. Conversely, higher levels of concern correspond to less favorable views. While these results derive from a Spanish sample, they align with international evidence reporting similar patterns in other educational contexts (Watkins et al., 2019; Yerliyurt Günay et al., 2023).

Our findings are consistent with previous international studies. For example, the greater attitudinal barriers found among Secondary Education teachers align with results from (Humphrey & Symes, 2013) in the UK. Similarly, the positive role of teacher training mirrors the evidence reported by (Crispel & Kasperski, 2021). The contrast between mainstream and special schools’ parallels findings from (Waddington & Reed, 2017) reinforcing the international relevance of our results.

One limitation of this study is that the use of a convenience sampling method restricts the generalizability of the findings. Second, socio-economic and institutional contextual variables (e.g., leadership styles, availability of resources) were not considered in depth, which may influence inclusive practices. Finally, the cross-sectional design limits causal interpretations. Future studies should adopt longitudinal approaches and incorporate contextual variables to provide a more comprehensive understanding. The originality of this study lies in the large and diverse national sample of 2310 teachers, representing all educational stages and school types in Spain, and in the combined use of the INDEX and INTEA-EDG instruments, which, to our knowledge, has not been previously applied in this context. These findings advance current understanding by identifying teacher profiles with greater attitudinal barriers, which can inform targeted training and intervention programs.

In conclusion, this study urges the implementation of practical training programs that increase opportunities for contact in supportive contexts. This would lead to more satisfactory experiences for teachers by providing them with concrete intervention and classroom adaptation strategies and skills.

## 5. Conclusions

This study demonstrates that teachers’ attitudes, feelings, and concerns are key determinants of the success of inclusive education for students with ASD. Significant differences emerged according to gender, school type, educational stage, specialty, and training hours. Overall, teachers with more training and those in early childhood and primary education showed more favorable perceptions, while secondary teachers expressed greater concerns. Specialists in educational guidance and attention to diversity reported more positive attitudes, highlighting the importance of specific preparation.

These findings stress the need to strengthen teacher education in inclusive pedagogy for all professionals, not only specialists, and to reinforce support structures within mainstream schools where diversity is most present. International comparisons suggest that continuous professional development, collaborative teaching models, and supportive institutional climates are universally effective in promoting positive attitudes toward inclusion. Future research should deepen the analysis of early educational stages and explore cross-cultural perspectives to further advance inclusive education.

## Figures and Tables

**Table 1:** Mann–Whitney U Test for Comparing Gender in the SACIE-R and INTEA Questionnaires.

**SACIE-R FACTOR 1: Attitudes**				
	**N**	**Middle Range**	**Mann–Whitney U Test**	**Sig (*p*)**
**Men**	459	1131.98	435,599	0.389
**Women**	1851	1161.33		
**SACIE-R FACTOR 2: Feelings**				
	**N**	**Middle Range**	**Mann–Whitney U Test**	**Sig (*p*)**
**Men**	459	1337.56	341,237.35	0.000
**Women**	1851	1110.35		
**SACIE-R FACTOR 3: Concerns**				
	**N**	**Middle Range**	**Mann–Whitney U Test**	**Sig (*p*)**
**Men**	459	1256.53	378,434	0.000
**Women**	1851	1130.45		
**INTEA FACTOR 1: Philosophical Issues**				
	**N**	**Middle Range**	**Mann–Whitney U Test**	**Sig (*p*)**
**Men**	459	1197.90	405,343	0.125
**Women**	1851	1144.99		
**INTEA FACTOR 2: Benefits of Inclusion**				
	**N**	**Middle Range**	**Mann–Whitney U Test**	**Sig (*p*)**
**Men**	459	1193.53	407,347	0.168
**Women**	1851	1146.07		
**Total INTEA Score**				
	**N**	**Middle Range**	**Mann–Whitney U Test**	**Sig (*p*)**
**Men**	459	1197.45	405,549.50	0.131
**Women**	1851	1145.10		

**Table 2:** ANOVA Test for comparing school type and the SACIE-R and INTEA questionnaires.

**SACIE-R FACTOR 1: Attitudes**				**ANOVA**	
	**N**	**Mean**	**Standard Deviation**	**F**	**Sig. (*p*)**
**Regular School**	1802	3.0585	0.55847	1.288	0.276
**Regular School with Special Education Classroom**	358	0.0145	0.55832		
**Special Education Center**	79	2.9949	0.69132		
**SACIE-R FACTOR 2: Feelings**				**ANOVA**	
	**N**	**Mean**	**Standard Deviation**	**F**	**Sig. (*p*)**
**Regular School**	1802	1.4286	0.44528	16.387	0.000
**Regular School with Special Education Classroom**	358	1.4115	0.43938		
**Special Education Center**	79	1.1392	0.29530		
**SACIE-R FACTOR 3: Concerns**				**ANOVA**	
	**N**	**Mean**	**Standard Deviation**	**F**	**Sig. (*p*)**
**Regular School**	1802	2.6580	0.63275	31.285	0.000
**Regular School with Special Education Classroom**	358	2.6732	0.63348		
**Special Education Center**	79	2.0918	0.49546		
**INTEA FACTOR 1: Philosophical Issues**				**ANOVA**	
	**N**	**Mean**	**Standard Deviation**	**F**	**Sig. (*p*)**
**Regular School**	1802	3.0518	0.55873	11.960	0.000
**Regular School with Special Education Classroom**	358	2.9101	0.54906		
**Special Education Center**	79	2.8861	0.67458		
**INTEA FACTOR 2: Benefits of Inclusion**				**ANOVA**	
	**N**	**Mean**	**Standard Deviation**	**F**	**Sig. (*p*)**
**Regular School**	1802	2.9875	0.53323	8.461	0.000
**Regular School with Special Education Classroom**	358	2.8866	0.51049		
**Special Education Center**	79	2.8177	0.64226		
**Total INTEA Score**				**ANOVA**	
	**N**	**Mean**	**Standard Deviation**	**F**	**Sig. (*p*)**
**Regular School**	1802	3.0196	0.51160	11.533	0.000
**Regular School with Special Education Classroom**	358	2.8983	0.48720		
**Special Education Center**	79	2.8519	0.63121		

**Table 3:** ANOVA Test for comparing educational stage and the SACIE-R and INTEA questionnaires.

**SACIE-R FACTOR 1: Attitudes**				**ANOVA**	
	**N**	**Mean**	**Standard Deviation**	**F**	**Sig. (*p*)**
**Early Childhood Education**	581	3.0317	0.58601	5.622	0.000
**Primary Education**	716	3.1201	0.55881		
**Secondary Education**	942	3.0121	0.54617		
**E.B.O**	43	2.8698	0.71831		
**T.V.A**	28	2.8857	0.66762		
**SACIE-R FACTOR 2: Feelings**				**ANOVA**	
	**N**	**Mean**	**Standard Deviation**	**F**	**Sig. (*p*)**
**Early Childhood Education**	581	1.3964	0.43619	5.019	0.000
**Primary Education**	716	1.3962	0.44277		
**Secondary Education**	942	1.4501	0.44756		
**E.B.O**	43	1.3178	0.41756		
**T.V.A**	28	1.1548	0.33311		
**SACIE-R FACTOR 3: Concerns**				**ANOVA**	
	**N**	**Mean**	**Standard Deviation**	**F**	**Sig. (*p*)**
**Early Childhood Education**	581	2.5654	0.62476	9.993	0.000
**Primary Education**	716	2.6009	0.62860		
**Secondary Education**	942	2.7264	0.63853		
**E.B.O**	43	2.4709	0.62012		
**T.V.A**	28	2.2946	0.55717		
**INTEA FACTOR 1: Philosophical Issues**				**ANOVA**	
	**N**	**Mean**	**Standard Deviation**	**F**	**Sig. (*p*)**
**Early Childhood Education**	581	3.0090	0.53838	1.522	0.193
**Primary Education**	716	3.0304	0.56084		
**Secondary Education**	942	3.0335	0.57565		
**E.B.O**	43	2.9023	0.62697		
**T.V.A**	28	2.8286	0.68792		
**INTEA FACTOR 2: Benefits of Inclusion**				**ANOVA**	
	**N**	**Mean**	**Standard Deviation**	**F**	**Sig. (*p*)**
**Early Childhood Education**	581	2.9559	0.51661	2.213	0.065
**Primary Education**	716	3.0045	0.52770		
**Secondary Education**	942	2.9493	0.54347		
**E.B.O**	43	2.8326	0.56049		
**T.V.A**	28	2.8500	0.64779		
**Total INTEA Score**				**ANOVA**	
	**N**	**Mean**	**Standard Deviation**	**F**	**Sig. (*p*)**
**Early Childhood Education**	581	2.9824	0.49039	1.736	0.139
**Primary Education**	716	3.0175	0.51298		
**Secondary Education**	942	2.9914	0.52275		
**E.B.O**	43	2.8674	0.56557		
**T.V.A**	28	2.8393	0.63732		

**Table 4:** ANOVA Test for comparing teaching specialty and the SACIE-R and INTEA questionnaires.

**SACIE-R FACTOR 1: Attitudes**				**ANOVA**	
	**N**	**Mean**	**Standard Deviation**	**F**	**Sig. (*p*)**
**Early Childhood Education Tutor**	180	2.8622	0.51276	17.297	0.000
**Primary Education Tutor**	231	3.0563	0.51494		
**Specialist in Early Childhood and Primary Education**	173	2.9642	0.50473		
**Specialist in Attention to Diversity**	493	3.1095	0.62392		
**Specialist in Educational Guidance**	353	3.2550	0.56732		
**Secondary School Teacher**	608	2.9368	0.54039		
**Management Team**	272	3.0713	0.53655		
**SACIE-R FACTOR 2: Feelings**				**ANOVA**	
	**N**	**Mean**	**Standard Deviation**	**F**	**Sig. (*p*)**
**Early Childhood Education Tutor**	180	1.4796	0.47555	13.795	0.000
**Primary Education Tutor**	231	1.4805	0.48403		
**Specialist in Early Childhood and Primary Education**	173	1.4836	0.43790		
**Specialist in Attention to Diversity**	493	1.2934	0.38605		
**Specialist in Educational Guidance**	353	1.3343	0.41856		
**Secondary School Teacher**	608	1.4918	0.45069		
**Management Team**	272	1.4167	0.43758		
**SACIE-R FACTOR 3: Concerns**				**ANOVA**	
	**N**	**Mean**	**Standard Deviation**	**F**	**Sig. (*p*)**
**Early Childhood Education Tutor**	180	2.7083	0.61663	47.678	0.000
**Primary Education Tutor**	231	2.7597	0.61385		
**Specialist in Early Childhood and Primary Education**	173	2.8353	0.57972		
**Specialist in Attention to Diversity**	493	2.3306	0.58994		
**Specialist in Educational Guidance**	353	2.4412	0.63227		
**Secondary School Teacher**	608	2.8664	0.59144		
**Management Team**	272	2.6563	0.58615		
**INTEA FACTOR 1: Philosophical Issues**				**ANOVA**	
	**N**	**Mean**	**Standard Deviation**	**F**	**Sig. (*p*)**
**Early Childhood Education Tutor**	180	2.8933	0.51715	13.502	0.000
**Primary Education Tutor**	231	2.9489	0.59387		
**Specialist in Early Childhood and Primary Education**	173	2.9202	0.56927		
**Specialist in Attention to Diversity**	493	3.0665	0.52680		
**Specialist in Educational Guidance**	353	3.2357	0.48427		
**Secondary School Teacher**	608	2.9740	0.60502		
**Management Team**	272	2.9787	0.56017		
**INTEA FACTOR 2: Benefits of Inclusion**				**ANOVA**	
	**N**	**Mean**	**Standard Deviation**	**F**	**Sig. (*p*)**
**Early Childhood Education Tutor**	180	2.8589	0.51250	16.137	0.000
**Primary Education Tutor**	231	2.9506	0.55622		
**Specialist in Early Childhood and Primary Education**	173	2.8301	0.54944		
**Specialist in Attention to Diversity**	493	3.0442	0.49246		
**Specialist in Educational Guidance**	353	3.1530	0.47881		
**Secondary School Teacher**	608	2.8674	0.55486		
**Management Team**	272	2.9610	0.52715		
**Total INTEA Score**				**ANOVA**	
	**N**	**Mean**	**Standard Deviation**	**F**	**Sig. (*p*)**
**Early Childhood Education Tutor**	180	2.8761	0.47430	16.237	0.000
**Primary Education Tutor**	231	2.9498	0.54580		
**Specialist in Early Childhood and Primary Education**	173	2.8751	0.52906		
**Specialist in Attention to Diversity**	493	3.0554	0.47537		
**Specialist in Educational Guidance**	353	3.1943	0.44155		
**Secondary School Teacher**	608	2.9207	0.54371		
**Management Team**	272	2.9699	0.50821		

**Table 5:** Correlation between training tours and the SACIE-R and INTEA questionnaires.

Spearman Correlation	Training Hours		
	N	Correlation Coefficient	Sig. (*p*)
**SACIE-R FACTOR 1: Attitudes**	2310	0.150	0.000
**SACIE-R FACTOR 2: Feelings**	2310	−0.180	0.000
**SACIE-R FACTOR 3: Concerns**	2310	−0.246	0.000
**INTEA FACTOR 1: Philosophical Issues**	2310	0.100	0.000
**INTEA FACTOR 2: Benefits of Inclusion**	2310	0.125	0.000
**Total INTEA Score**	2310	0.123	0.000

**Table 6:** Correlation between SACIE-R and INTEA questionnaires.

Spearman Correlation	SACIE-R FACTOR 1: Attitudes			SACIE-R FACTOR 2: Feelings			SACIE-R FACTOR 3: Concerns		
	N	Correlation Coefficient	Sig. (*p*)	N	Correlation Coefficient	Sig. (*p*)	N	Correlation Coefficient	Sig. (*p*)
**INTEA FACTOR 1: Philosophical Issues**	2310	0.498	0.000	2310	−0.221	0.000	2310	−0.213	0.000
**INTEA FACTOR 2: Benefits of Inclusion**	2310	0.459	0.000	2310	−0.223	0.000	2310	−0.224	0.000
**Total INTEA Score**	2310	0.515	0.000	2310	−0.244	0.000	2310	−0.233	0.000

## Data Availability

Data is contained within the article.

## Supplementary Materials

The following supporting information can be downloaded at https://www.mdpi.com/article/10.3390/ejihpe15100200/s1, Supplementary S1: Questionnaires used in the research.

## Appendix A

- AREISA

- SACIE-R

SACIE-R SCALE (Forlin et al., 2011; adapted by Fuentes et al., 2019).

Part II. Feelings, Attitudes and Concerns about Inclusive Education.

Below is a series of statements related to inclusive education, which involves a wide range of students with diverse backgrounds and abilities, learning alongside their peers in regular schools. Please indicate the degree of agreement or disagreement with each statement using the following scale:

**1 Strongly disagree**	**2 Disagree**	**3 Agree**	**4 Strongly agree**

**1. Students with difficulties in oral expression should be in regular classes.**	1	2	3	4
**2. I find it difficult to give adequate attention to all students in a classroom.**	1	2	3	4
**3. I tend to end my contacts with people with disabilities as soon as possible.**	1	2	3	4
**4. Students with attention problems should be in regular classes.**	1	2	3	4
**5. I am concerned that my workload will increase by having students with disabilities in my class.**	1	2	3	4
**6. Students who use alternative and/or augmentative communication systems (e.g., Braille/sign language) should be in regular classes.**	1	2	3	4
**7. I am concerned about being more stressed by having students with disabilities in my class.**	1	2	3	4
**8. I am afraid of looking directly at a person with a disability.**	1	2	3	4
**9. Students who frequently fail subjects should be in regular classes.**	1	2	3	4
**10. I find it difficult to overcome the impression I get when meeting people with severe physical disabilities.**	1	2	3	4
**11. I am concerned about not having the necessary knowledge and skills to teach students with disabilities.**	1	2	3	4
**12. Students who need an individualized academic program should be in regular classes.**	1	2	3	4

- INTEA

**1. As a teacher, I believe that inclusion is the most appropriate way to serve students with autism.**	1	2	3	4
**2. Students with autism have the right to receive their entire education within the regular classroom.**	1	2	3	4
**3. The inclusion of children with autism in the classroom will benefit their peers, as they will learn to work and live together with people with functional diversity.**	1	2	3	4
**4. The extra attention required by a child with autism will not negatively affect their classmates in the normal development of the teaching-learning process.**	1	2	3	4
**5. All teachers should receive training and have sufficient knowledge to adequately address the special educational needs of children with autism.**	1	2	3	4
**6. A student with autism in the regular classroom will improve their performance as a result of their inclusion.**	1	2	3	4
**7. The behavior of a student with autism can be successfully managed within the regular classroom.**	1	2	3	4
**8. I believe there are enough personal and material resources in classrooms to support students with autism.**	1	2	3	4
**9. If I had a student with autism in my classroom, I would not feel comfortable and would prefer that they attend a special education center or spend as much time as possible with the school specialists.**	1	2	3	4
**10. As a teacher, I have the knowledge and skills to adequately teach a student with autism.**	1	2	3	4
**11. Students with autism will develop social skills as a result of their inclusion in the regular classroom.**	1	2	3	4
**12. The inclusion of students with autism in the classroom will have a positive impact on the academic performance of my other students.**	1	2	3	4
**13. The school management team, the administration, and the teachers provide a supportive and collaborative environment that fosters inclusive education in classrooms.**	1	2	3	4
**14. It is more difficult to maintain appropriate classroom behavior when also including students with autism.**	1	2	3	4
**15. It is more difficult to maintain appropriate classroom behavior when also including students with autism.**	1	2	3	4
**16. I believe I can collaborate effectively with other people (doctors, specialists, families, TSS, TIS, etc.) to meet the needs of a child with autism in my regular classroom.**	1	2	3	4
**17. The behavior of the class group will be a positive example for the student with autism in my classroom.**	1	2	3	4
**18. If I have a student with autism in my regular classroom, I believe that coordination and regular meetings with the school management team, other teachers, and parents are essential to facilitate the teaching-learning process.**	1	2	3	4
**19. Students with autism will achieve greater self-esteem as a result of being included in the regular classroom.**	1	2	3	4
**20. The inclusion of a student with autism in the regular classroom will not require significant changes to the class pace.**	1	2	3	4
**21. Students with autism will initiate more interactions with their peers and teachers as a result of being included in the regular classroom.**	1	2	3	4
**22. The inclusion of students with autism in the regular classroom does not affect the performance of the rest of the group.**	1	2	3	4
