# Supplementary material for: The Educational Inclusion of Students with Autism Spectrum Disorder: Teachers’ Feelings, Attitudes, and Concerns About Inclusion in Spain
Source: Eur J Investig Health Psychol Educ. 2025 Sep 29;15(10):200. doi: 10.3390/ejihpe15100200 (PMC12563666; doi:10.3390/ejihpe15100200)
Supplement: Supplementary file 1 [file ejihpe-15-00200-s001.zip › ejihpe-3767737-supplementary.pdf]

European Journal of
Investigation in Health,
Psychology and Education

MDPI

Supplementary S1

- AREISA

Attitudes of Regular Educators toward the Inclusion of Students with Autism Spectrum Disorder

This survey investigates the attitudes of regular educators toward inclusion for students with autism spectrum disorder. For the purposes of this survey, autism spectrum disorder encompasses Pervasive Developmental Disorder, Asperger's Disorder, and Autism. Indicate your response which most closely reflects your agreement or disagreement with the each statement. Completion of this survey should take approximately 10-15 minutes. There are no correct or incorrect answers.

Demographic Information

Gender	Male ☐	Female ☐	
Years of Experience (Including this year)	0-5 ☐	6-15 ☐	16+ ☐
Current teaching placement	K-5 ☐	6-8 ☐	9-12 ☐
Do you have previous experience with including child with a disability in your regular education classroom?	Yes ☐	No ☐	
Amount of training regarding autism (in hours)	0 ☐	1-5 ☐	6-10 ☐
	11+ ☐		

	Strongly Disagree -2	Disagree -1	Agree +1	Strongly Agree +2
1 As a regular educator, I believe that inclusion is the most appropriate way to service students with autism.	O	O	O	O
2 Students with autism have the right to receive all education within the regular education classroom.	O	O	O	O
3 Including students with autism will benefit typical students as they will learn to accept students with disabilities.	O	O	O	O
4 The extra attention that will have to be given to a student with autism will not take away from the education of the other students.	O	O	O	O
5 My principal and school administrators promote the philosophy that students with disabilities are the responsibility of all school personnel.	O	O	O	O
6 A student with autism included in the regular classroom will display academic gains as a result of being included.	O	O	O	O
7 The behavior of a student with autism can be successfully managed within the regular education classroom.	O	O	O	O
8 There are enough resources (materials, personnel) in place to support a student with autism being placed in my regular education classroom.	O	O	O	O
9 Should a student with autism be placed in my classroom, my administrators would provide time for regular education staff and special education staff to discuss and plan for the student.	O	O	O	O
10 Regular educators possess the knowledge and skills to adequately teach a child with autism.	O	O	O	O
11 The student with autism will develop social skills as a result of being included within the regular education classroom.	O	O	O	O
12 Including students with autism in the regular education classroom will positively impact the academic achievement of typical students.	O	O	O	O
13 I believe that my principal and other administrators provide a supportive, collaborative environment that is conducive to providing inclusive education.	O	O	O	O
14 It will not be more difficult to maintain appropriate classroom behavior when a student with autism is included in my classroom.	O	O	O	O
15 I am knowledgeable regarding curriculum modifications that have proven helpful in teaching a child with autism spectrum disorder.	O	O	O	O
16 I believe that I can collaborate effectively with other staff to meet the needs of a child with autism included in my classroom.	O	O	O	O
17 The behavior of the regular education students will set a positive example for the autistic student included in the regular education classroom.	O	O	O	O
18 Should a child with autism be placed within my regular education classroom, I believe that my principal would periodically check in to see if assistance is necessary.	O	O	O	O
19 The student with autism will possess an increased self esteem as a result of being included within the regular education classroom.	O	O	O	O
20 Including a student with autism in the regular education classroom will not require significant changes in pacing so that I can still meet the district benchmarks within the required times.	O	O	O	O
21 The student with autism will initiate more interactions with peers and teachers as a result of being included within the regular education classroom.	O	O	O	O
22 Standardized test scores will not be affected by the inclusion of students with autism in the regular education classroom.	O	O	O	O

- SACIE-R

Anexo I. Versión final del instrumento para población española

ESCALA SACIE-R (Forlin et al., 2011; adaptada por Rodríguez, Caurcel y Alaín)
II Parte. Sentimientos, Actitudes y preocupaciones acerca de la Educación Inclusiva
A continuación se presenta una serie de afirmaciones relativas a la educación inclusiva, la cual involucra a una amplia gama de estudiantes con antecedentes y habilidades diversas, que aprenden con sus compañeros en escuelas regulares. Por favor, indique el grado de acuerdo o desacuerdo con las mismas, utilizando la siguiente escala:

1 Totalmente en desacuerdo	2 En desacuerdo	3 De acuerdo	4 Totalmente de acuerdo

1. El alumnado con dificultades para expresarse oralmente debería estar en clases regulares	1	2	3	4
2. Considero que es difícil prestar una atención adecuada a todos los estudiantes en un aula	1	2	3	4
3. Tiendo a finalizar mis contactos con personas con discapacidad tan pronto como sea posible	1	2	3	4
4. El alumnado con problemas de atención debe estar en clases regulares	1	2	3	4
5. Me preocupa que mi carga de trabajo se vea incrementada por tener alumnado con discapacidad en mi clase	1	2	3	4
6. El alumnado que utiliza sistemas de comunicación alternativos y/o aumentativos (por ejemplo, Braille / lenguaje de signos) debería estar en clases regulares	1	2	3	4
7. Me preocupa estar más estresado por tener alumnado con discapacidad en mi clase	1	2	3	4
8. 11. Me da miedo mirar directamente a una persona con discapacidad	1	2	3	4
9. El alumnado que suspende asignaturas frecuentemente debe estar en clases regulares	1	2	3	4
10. Me resulta difícil superar la impresión que me produce conocer a personas con graves discapacidades físicas	1	2	3	4
11. Me preocupa no tener los conocimientos y habilidades necesarios para enseñar al alumnado con discapacidad	1	2	3	4
12. El alumnado que necesita un programa académico individualizado debe estar en clases regulares	1	2	3	4

- **INTEA**

1. Escala Likert de 4 puntos
2. Totalmente en desacuerdo/2 en desacuerdo/ 3 de acuerdo / 4 totalmente de acuerdo
3. Como docente creo que la inclusión es la forma más adecuada de atender a los estudiantes con autismo.
4. Los estudiantes con autismo tienen derecho a recibir toda la educación dentro del aula ordinaria.
5. La inclusión de los niños y niñas con autismo en el aula beneficiará a sus compañeros y compañeras, ya que estos aprenderán a trabajar y convivir con personas con diversidad funcional.
6. La atención extra que requiere un niño o niña con autismo no perjudicará al resto de sus compañeros y compañeras en el normal desarrollo del proceso de enseñanza-aprendizaje.

7. Todos los docentes deberán formarse y tener los conocimientos suficientes para poder atender adecuadamente las necesidades educativas especiales de los niños y niñas con autismo.
8. Un estudiante con autismo en el aula ordinaria mejorará su rendimiento como resultado de su inclusión.
9. El comportamiento de un estudiante con autismo se puede manejar con éxito dentro del aula ordinaria.
10. Creo que hay suficientes recursos personales y materiales en las clases para atender a los estudiantes con autismo.
11. Si tuviera un alumno o alumna con autismo en mi aula no me sentiría cómodo/a y preferiría que acudiera a un centro de educación especial o saliera con los especialistas del colegio el mayor tiempo posible.
12. Como docente tengo el conocimiento y las habilidades para enseñar adecuadamente a un alumno o alumna con autismo.
13. El alumnado con autismo desarrollarán habilidades sociales como resultado de su inclusión en el aula ordinaria.
14. La inclusión de estudiantes con autismo en el aula tendrá un impacto positivo en el rendimiento académico del resto de mis alumnos y alumnas.
15. El equipo directivo, la Administración y los docentes brindan un entorno de apoyo y colaboración que son propicios para fomentar la educación inclusiva en las aulas.
16. Es más difícil mantener un comportamiento apropiado en el aula cuando se trabaja también incluyendo a alumnado con autismo.
17. Tengo conocimiento sobre las modificaciones en el currículo y las metodologías educativas que han demostrado ser útiles para enseñar a un niño o niña con trastorno del espectro autista.
18. Creo que puedo colaborar eficazmente con otras personas (médicos, especialistas, familias, TSS, TIS…) para satisfacer las necesidades de un niño con autismo de mi aula ordinaria.
19. El comportamiento del grupo-clase será un ejemplo positivo para el estudiante con autismo de mi aula.
20. Si tengo un alumno/a con autismo en mi aula ordinaria, creo que es esencial la coordinación y las reuniones periódicas tanto con el equipo directivo como con el resto de docentes y con los padres para facilitar el proceso de enseñanza-aprendizaje.
21. El alumnado con autismo alcanzará una mayor autoestima como resultado de ser incluido en el aula ordinaria.
22. La inclusión de un alumno/a con autismo en el aula ordinaria no requerirá cambios significativos en el ritmo de la clase.
23. El alumnado con autismo iniciará más interacciones con sus compañeros y compañeras y docentes como resultado de estar incluido dentro del aula ordinaria.
24. La inclusión de alumnado con autismo en el aula ordinaria no afecta al rendimiento del resto del grupo.
